# Comparative Uptake Patterns of Radioactive Iodine and [18F]-Fluorodeoxyglucose (FDG) in Metastatic Differentiated Thyroid Cancers

**DOI:** 10.3390/jcm13133963

**Published:** 2024-07-06

**Authors:** Devan Diwanji, Emmanuel Carrodeguas, Youngho Seo, Hyunseok Kang, Myat Han Soe, Janet M. Chiang, Li Zhang, Chienying Liu, Spencer C. Behr, Robert R. Flavell

**Affiliations:** 1 Department of Radiology and Biomedical Imaging, University of California, San Francisco, CA 94143, USA; devan.diwanji@ucsf.edu (D.D.); emmanuel.carrodeguas@ucsf.edu (E.C.); youngho.seo@ucsf.edu (Y.S.); spencer.behr@ucsf.edu (S.C.B.); 2 Medical Scientist Training Program, University of California, San Francisco, CA 94143, USA; 3 Molecular Biophysics and Integrated Bioimaging Division, Lawrence Berkeley National Laboratory, Berkeley, CA 94720, USA; 4 Joint Graduate Group in Bioengineering, University of California, San Francisco, CA 94720, USA; 5 Department of Nuclear Engineering, University of California, Berkeley, CA 94720, USA; 6 Division of Hematology/Oncology, Department of Medicine, University of California, San Francisco, CA 94143, USA; hyunseok.kang@ucsf.edu (H.K.); li.zhang@ucsf.edu (L.Z.); 7 Division of Endocrinology, Department of Medicine, University of California, San Francisco, CA 94143, USA; myat.soe@ucsf.edu (M.H.S.); janet.chiang@ucsf.edu (J.M.C.); chienying.liu@ucsf.edu (C.L.); 8 Division of Endocrinology, Department of Medicine, San Francisco VA Healthcare System, San Francisco, CA 94121, USA; 9 Division of Endocrinology, Department of Medicine, Zuckerberg San Francisco General Hospital and Trauma Center, San Francisco, CA 94110, USA; 10 Department of Epidemiology and Biostatistics, University of California, San Francisco, CA 94143, USA; 11 Molecular Imaging and Therapeutics Clinical Section, Department of Radiology and Biomedical Imaging, University of California, San Francisco, CA 94143, USA; 12 Department of Pharmaceutical Chemistry, University of California, San Francisco, CA 94143, USA

**Keywords:** thyroid cancer, RAI, Iodine-131, positron emission tomography, PET/CT, PET, differentiated thyroid cancer

## Abstract

**Background:** Metastatic differentiated thyroid cancer (DTC) represents a molecularly heterogeneous group of cancers with varying radioactive iodine (RAI) and [^18^F]-fluorodeoxyglucose (FDG) uptake patterns potentially correlated with the degree of de-differentiation through the so-called “flip-flop” phenomenon. However, it is unknown if RAI and FDG uptake patterns correlate with molecular status or metastatic site. **Materials and Methods:** A retrospective analysis of metastatic DTC patients (n = 46) with radioactive 131-iodine whole body scan (WBS) and FDG-PET imaging between 2008 and 2022 was performed. The inclusion criteria included accessible FDG-PET and WBS studies within 1 year of each other. Studies were interpreted by two blinded radiologists for iodine or FDG uptake in extrathyroidal sites including lungs, lymph nodes, and bone. Cases were stratified by *BRAF* V600E mutation status, histology, and a combination of tumor genotype and histology. The data were analyzed by McNemar’s Chi-square test. **Results:** Lung metastasis FDG uptake was significantly more common than iodine uptake (WBS: 52%, FDG: 84%, *p* = 0.04), but no significant differences were found for lymph or bone metastases. Lung metastasis FDG uptake was significantly more prevalent in the papillary pattern sub-cohort (WBS: 37%, FDG: 89%, *p* = 0.02) than the follicular pattern sub-cohort (WBS: 75%, FDG: 75%, *p* = 1.00). Similarly, *BRAF* V600E+ tumors with lung metastases also demonstrated a preponderance of FDG uptake (WBS: 29%, FDG: 93%, *p* = 0.02) than *BRAF* V600E− tumors (WBS: 83%, FDG: 83%, *p* = 1.00) with lung metastases. Papillary histology featured higher FDG uptake in lung metastasis (WBS: 39%, FDG: 89%, *p* = 0.03) compared with follicular histology (WBS: 69%, FDG: 77%, *p* = 1.00). Patients with papillary pattern disease, *BRAF* V600E+ mutation, or papillary histology had reduced agreement between both modalities in uptake at all metastatic sites compared with those with follicular pattern disease, *BRAF* V600E− mutation, or follicular histology. Low agreement in lymph node uptake was observed in all patients irrespective of molecular status or histology. **Conclusions:** The pattern of FDG-PET and radioiodine uptake is dependent on molecular status and metastatic site, with those with papillary histology or *BRAF* V600E+ mutation featuring increased FDG uptake in distant metastasis. Further study with an expanded cohort may identify which patients may benefit from specific imaging modalities to recognize and surveil metastases.

## 1. Introduction

Differentiated thyroid cancers (DTCs) consists of histologically and, more recently, molecularly defined variants [1,2,3,4]. Nearly 10–30% of DTCs metastasize or recur, and up to 50% of metastatic cases are refractory to the standard radioactive iodine (RAI) therapy [5,6,7]. Metastatic DTCs carry a 60% 5-year survival compared with 99% in patients without metastases [8]. Identifying which patients may benefit from RAI or alternative therapies may improve patient outcomes. 

Imaging with ^18^F-Fluorodeoxyglucose (FDG) positron emission technology (PET) and RAI whole body scan (WBS) play an important role in the identification, staging, and assessment of treatment response in metastatic DTCs. Previous studies have shown that FDG positivity correlates with a worse prognosis irrespective of RAI uptake and that FDG uptake is often inversely correlated with RAI uptake through the “flip-flop phenomenon” [9,10,11,12,13,14]. The flip-flop phenomenon observed in thyroid and certain neuroendocrine malignancies describes an imaging finding in which the uptake of a tissue-specific tracer (RAI for thyroid) is inversely related to the uptake of a non-tissue-specific tracer (FDG, for example) [15,16]. Although the molecular basis of the flip-flop phenomenon remains under investigation, FDG uptake in DTCs may serve as a marker for tumor de-differentiation, increased aggressiveness, and decreased responsiveness to empiric RAI therapy [12,17,18,19,20].

Recent efforts in uncovering the molecular pathogenesis of DTCs have led to the identification oncogenic driver mutations and enabled precise therapeutic targeting. Histologic classification schemes have been supplemented by the presence or absence of mutations in oncogenes such as *BRAF*, *RAS*, and *RET* [4,21]. For example, papillary thyroid cancers (PTCs) constituting nearly 80% of DTC cases are now divided into *BRAF* V600E-like (BVL) or *RAS*-like (RL) tumors with notable differences between the two groups [4,21,22,23]. BVL tumors which signal through mitogen-activated protein kinase (*MAPK*) pathways are more de-differentiated and associated with RAI refractoriness compared with RL tumors [24,25,26,27,28,29]. Studies have shown that redifferentiation by kinase specific inhibitors restores RAI sensitivity [30,31,32,33,34,35]. Furthermore, *TERT* promoter mutations are associated with more clinically aggressive disease independent of *BRAF* status [36].

Understanding the flip-flop phenomenon may help guide the imaging modality used to surveil metastases or recurrence as well as identify DTC patients who are refractory to RAI treatment and who could benefit from alternative therapies. Although molecular subtype closely relates to tumor dedifferentiation, no study to date has elucidated the relationship between molecular subtype and the flip-flop phenomenon at specific metastatic sites which may facilitate the appropriate selection of imaging modality. In this study, we investigated the flip-flop phenomenon in metastatic DTCs based on molecular subtype (presence or absence of *BRAF* V600E), histologic classification (papillary or follicular), a combination of molecular and histologic data (papillary pattern and follicular pattern), and metastatic site (lymph node, lung, and bone). 

## 2. Materials and Methods

### 2.1. Cohort Selection

A retrospective, IRB-approved (Protocols #10-02005 and 11-05345) study was performed at the University of California, San Francisco (UCSF) with patients diagnosed with biopsy-confirmed metastatic DTC who had both radioactive iodine WBS and FDG-PET within 12 months of each other. A 12-month imaging timeframe was chosen to limit the effects of potential tumor evolution between imaging studies without sacrificing statistical power. WBS imaging was conducted with I-131 post-therapy scans after either rhTSH (recombinant human thyroid stimulating hormone) or thyroid hormone withdrawal. Imaging studies were conducted from November 2008 to March 2022. Patients who received interval surgical, RAI, or medical management for thyroid cancer were excluded from the study. A total of 46 patients meeting the inclusion criteria were analyzed in the study (Table 1). Per patient data including histologic classification, classification, tumor genetics, and consensus RAI or FDG uptake are presented in Supplemental Table S1.

### 2.2. Histology and Molecular Data Classification

Histology and molecular data of the primary tumor, if available, were obtained through chart review. A total of 24 patients (52%) had papillary histology (13 classical, 4 Tall, and 1 sclerosing subtypes with 6 unknown subtypes), and 22 patients (48%) had follicular histology (including 10 with follicular thyroid cancer, 9 papillary cancer with follicular-like architecture (PTC-FV), and 3 with mixed papillary and follicular features) (Table 1). Patients with Hurthle cell histology were excluded from this analysis given the insufficient cohort size (n = 3) and molecular signatures distinct from standard follicular or papillary-like metastatic DTC [37].

A total of 29 (63%) patients had explicit *BRAF* testing, while 17 (37%) did not. Of those with *BRAF* testing, 20 patients (69%) were *BRAF*+, while 9 patients (31%) were *BRAF*−. For patients in whom *BRAF* testing was not available, it was presumed that either the test was performed but the results were not available upon chart review, or testing was not performed with a histopathologic diagnosis. A subset of patients (11 patients, 24% of entire cohort) also received tissue genomic panel analysis (UCSF500) [2,24,26] (Table 1). *RAS* and *TERT* promoter mutation status were obtained through tissue genomic panel testing and/or chart review.

Tumors were classified as papillary pattern (PP) or follicular pattern (FP) to integrate molecular and histologic tumor features. Papillary pattern tumors met at least one of the following criteria: (1) *BRAF* V600E+ mutation or *BRAF* fusion, (2) presence of *RET* fusion, (3) confirmed *RAS* negative mutation, or (4) papillary histology (excluding PTC-FV or mixed subtypes). Tumors in the papillary pattern sub-cohort most closely resemble *BRAF* V600E-like (BVL) tumors given that 20/27 (74%) featured *BRAF* V600E mutation or *RET* fusion [4]. Follicular pattern tumors met at least one of the following criteria: (1) presence of *RAS* mutation, (2) presence of BRAF mutation other than V600E or other mutation consistent with *RAS*-like axis dependence, or (3) PTC-FV or follicular histology. Tumors in the follicular pattern sub-cohort most closely resemble RAS-like (RL) tumors. If molecular testing and DTC subtype were unavailable, the primary histology was used to classify the tumor into papillary or follicular pattern. For example, DTCs from Patient ID 10 and 15 (see Supplementary Table S1) were classified as papillary pattern tumors given that only papillary histology was present in the pathology report and molecular testing was not available. A total of 27 patients (59% of entire cohort) were classified with papillary pattern tumors and 19 with follicular pattern tumors (41% of entire cohort).

## 3. Image Review

Each imaging study was independently read by two radiologists (E.C., a fellow with four years of radiology experience and one year of dedicated NM training, and S.C.B., a nuclear radiologist with 1 year of dedicated NM fellowship training and 10 years of independent practice), blinded to molecular status and histology. Each study was assessed for the presence of radiotracer uptake (qualitatively apparent uptake above background blood pool SUV_max_) on a regional basis in the lung, lymphatic tissue, and bone. Uptake in other organs was not assessed. Discordant reads were decided by a third blinded radiologist (R.R.F., a nuclear radiologist with 1 year of fellowship training and 6 years of dedicated NM practice). Consensus uptake at each site for both modalities is provided in Supplementary Table S1.

### 3.1. Statistical Analyses

McNemar’s Chi-square analysis in R (v4.0.5) was used to compare WBS and FDG uptake between molecular or histologic sub-cohorts. Statistical significance was defined as a two-sided alpha <0.05.

### 3.2. Agreement Analyses

Agreement between imaging modalities was assessed on an individual patient level by calculating the proportion of discordant uptake patterns in patients that had positive uptake by at least one modality at a particular metastatic site. Patients who did not have uptake at a particular site by either WBS or FDG were excluded from the analysis. Higher discordant rates indicate reduced agreement between imaging modalities.

## 4. Results

### 4.1. The Flip-Flop Phenomenon Varies by Metastatic Site and Molecular Status

We assessed for radiotracer uptake at three extra-thyroidal metastatic sites: lymphatic tissue, lung, and bone. In this cohort, 24 patients (52%) had lymph node uptake by either RAI or FDG, 31 (67%) had lung uptake, and 16 (35%) had bone uptake (Table 1).

**Figure 1:**
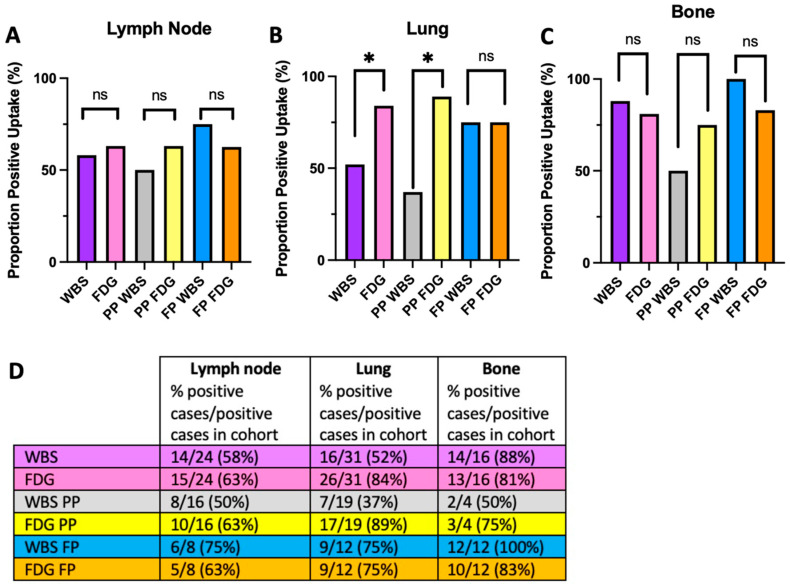
WBS and FDG uptake stratified by molecular subtype. WBS: radioactive iodine whole body scan; FDG: ^18^Fluorodeoxyglucose-PET; PP: papillary pattern; FP: follicular pattern. (**A**) No significant differences were observed in lymphatic tissue metastatic radiotracer uptake in any cohort. (**B**) Significantly increased FDG over iodine uptake was observed in the entire cohort in lung tissue and papillary pattern (PP) sub-cohort, but not in the follicular pattern (FP) sub-cohort. (**C**) No significant differences in radiotracer uptake were observed in bone in any cohort. (**D**) Table summary with proportions of patients which have metastases at any given site and proportions of patients that had FDG or WBS positivity. Asterisk (*) indicates *p* < 0.05; ns = not significant.

When considering lymph node uptake, no significant differences were observed in the entire cohort (WBS: n = 14/24, 58%; FDG: n = 15/24, 63%; *p* = 1.00), nor in the PP (WBS: n = 8/16, 50%; FDG: n = 10/16, 63%; p0.79) or FP (WBS: n = 6/8, 75%; FDG: n = 5/8, 63%; *p* = 1.00) sub-cohorts (Figure 1A,D). With respect to lung, significant differences in uptake were observed when considering the entire cohort (WBS: n = 16/31, 52%; FDG: n = 26/31, 84%; *p* = 0.04) supporting the flip-flop phenomenon at distant metastatic sites (Figure 1B,D). However, differences in uptake were maintained only in the PP sub-cohort (WBS: n = 7/19, 37%; FDG: n = 17/19, 89%; *p* = 0.02) and not in the FP sub-cohort (WBS: n = 9/12, 75%; FDG: n = 9/12, 75%; *p* = 1.00) (Figure 1B,D).

Like lymphatic tissue, no significant differences in bone uptake were observed in the entire cohort (WBS: n = 14/16, 88%; FDG: n = 13/16, 81%; *p* = 1.00), nor PP (WBS: n = 2/4, 50%; FDG: n = 3/4, 75%; *p* = 1.00) or FP sub-cohorts (WBS: n = 12/12, 100%; FDG: n = 10/12, 83%; *p* = 0.48) (Figure 1C,D). The flip-flop phenomenon is therefore not as evident in lymph or bone metastatic sites as it is in lung.

**Figure 2:**
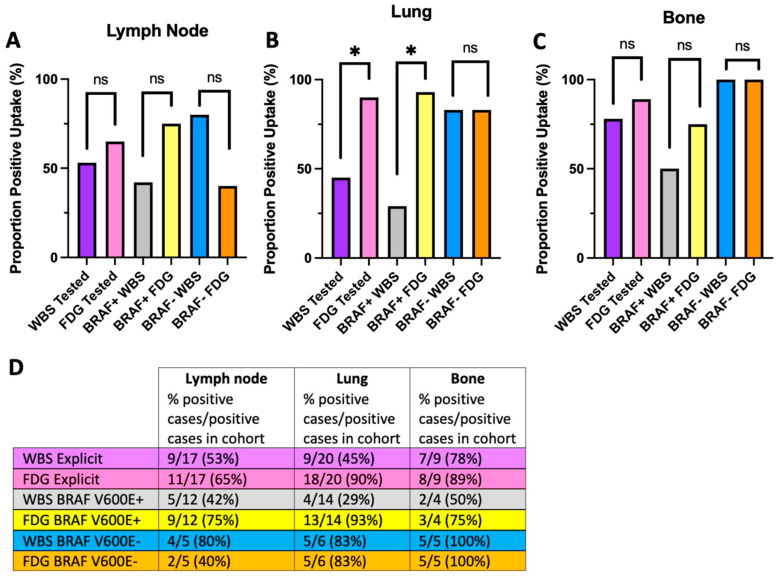
WBS and FDG uptake stratified by *BRAF* V600E status. WBS: radioactive iodine whole body scan; FDG: ^18^Fluorodeoxyglucose-PET. Only patients with *BRAF* testing are included in the cohort analysis (purple and pink for “WBS Tested” and “FDG Tested”, respectively). (**A**) No significant differences were observed in lymphatic tissue metastatic radiotracer uptake in any cohort. (**B**) Significantly increased FDG over iodine uptake was observed in the entire *BRAF* tested cohort in lung tissue and *BRAF+* sub-cohort, but not in the *BRAF*− sub-cohort. (**C**) No significant differences in radiotracer uptake were observed in bone in any cohort. (**D**) Table summary with proportions of patients which have metastases at any given site and proportions of patients that had FDG or WBS positivity. Asterisk (*) indicates *p* < 0.05; ns = not significant.

**Figure 3:**
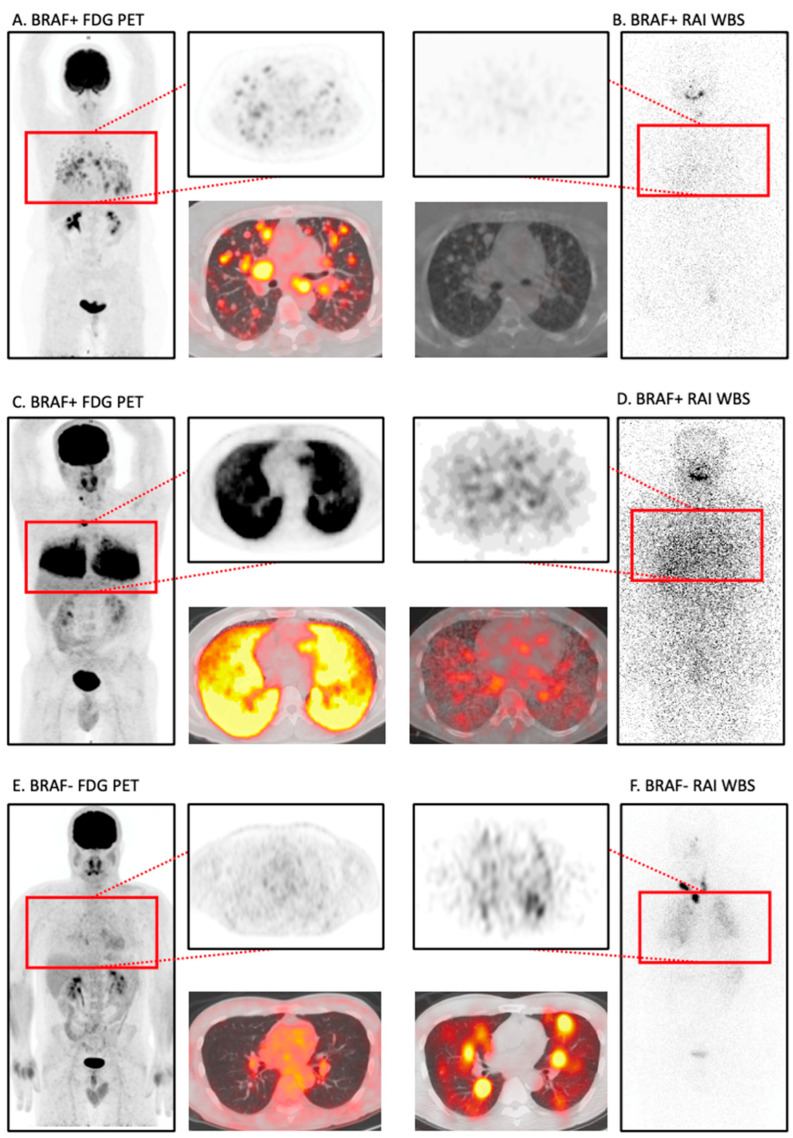
Example WBS and FDG PET scans of DTC lung metastasis. RAI WBS: radioactive iodine whole body scan; FDG: ^18^Fluorodeoxyglucose-PET. (**A**,**B**) Top row images are exemplary of DTC lung metastasis with FDG > WBS uptake in a patient with *BRAF+* disease, illustrative of the “flip-flop” phenomenon in a less differentiated tumor. Despite clear focal FDG avidity, iodine radiotracer is at background levels in WBS. (**C**,**D**) Middle row images demonstrate a concordant *BRAF+* case with iodine and FDG radiotracer avidity in the lung. (**E**,**F**) Bottom row images demonstrate *a BRAF*− case showing both low level FDG and iodine avidity in lung metastasis, both clearly above background levels, although more conspicuous by WBS.

We repeated the analysis excluding patients without *BRAF* testing (Figure 2). A similar pattern emerges in which *BRAF* V600E+ tumors demonstrate robust FDG uptake in lung metastases (WBS: n = 4/14, 29%; FDG: n = 13/14, 93%; *p* = 0.02) compared with that of *BRAF* V600E− tumors (WBS: n = 5/6, 83%; FDG: n = 5/6, 83%; *p* = 1.00) (Figure 2B,D). Example WBS and FDG images from patients with lung metastasis in *BRAF* V600E+ and *BRAF* V600E− disease are shown in Figure 3. 

*TERT* promoter molecular testing was also available for a subset of patients (n = 14, 30% of entire cohort). A total of 10 patients (22% of entire cohort) tested positive for mutations in the *TERT* promotor (*TERT+*), while 4 tested did not have *TERT* promoter mutations (*TERT*−). No differences between *TERT* sub-cohorts were noted in lymph nodes (*TERT*+ WBS: n = 4/6, 67%; FDG: n = 4/6, 67%; *p* = 1.00) (*TERT*− WBS: n = 1/1, 100%; FDG: n = 0/1, 0%; *p* = 1.00) or bone (*TERT*+ WBS: n = 3/5, 60%; FDG: n = 5/5, 100%; *p* = 0.48) (*TERT*− WBS: n = 1/1, 100%; FDG: n = 1/1, 100%; *p* = 1.00), although our results are limited by the small sample size (Supplementary Figure S1A,C,D). Like *BRAF* V600E+ and papillary pattern tumors, *TERT+* tumors exhibited FDG avidity in the lung (WBS: n = 4/8, 50%; FDG: n = 8/8, 100%; *p* = 0.08) compared with TERT− tumors (WBS: n = 3/3, 100%; FDG: n = 2/3, 67%; *p* = 1.00) (Supplementary Figure S2B,D).

### 4.2. The Flip-Flop Phenomenon and Histologic Classification

**Figure 4:**
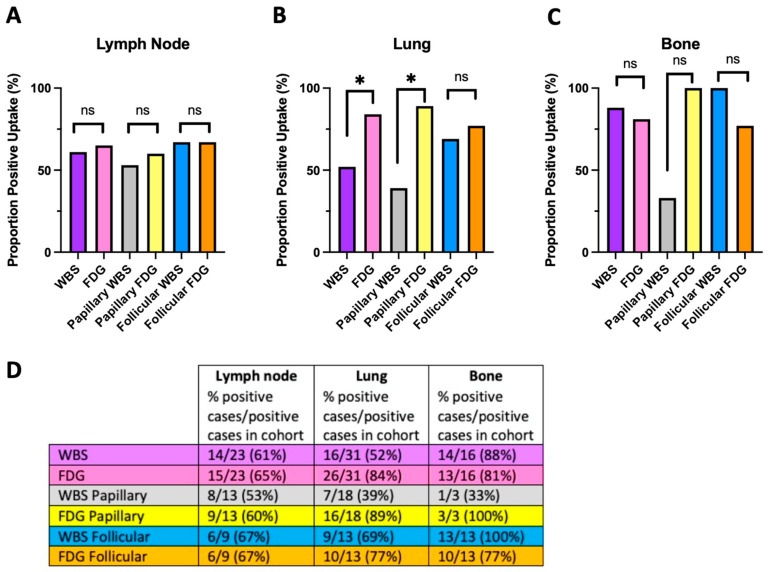
WBS and FDG uptake stratified by histology. (**A**) No significant differences were observed in lymphatic tissue metastatic radiotracer uptake in any cohort. (**B**) Significantly increased FDG over iodine uptake was observed in the entire cohort in lung tissue and papillary sub-cohort, but not in the follicular sub-cohort. (**C**) No significant differences in radiotracer uptake were observed in bone in any cohort. (**D**) Table summary with proportions of patients which have metastases at any given site and proportions of patients that had FDG or WBS positivity. Asterisk (*) indicates *p* < 0.05; ns = not significant.

We next subdivided the cohort by tumor histologic classification instead of molecular status. Radiotracer uptake in the lymphatic tissues remained similar in both imaging modalities across follicular (WBS: n = 6/9, 67%; FDG: n = 6/9, 67%; *p* = 1.00) and papillary (WBS: n = 8/15, 53%; FDG: n = 9/15, 60%; *p* = 1.00) tumor histology (Figure 4A,D). In the lung, primary tumors with follicular histology did not show differences in radiotracer uptake (WBS: n = 9/13, 69%; FDG: n = 10/13, 77%; *p* = 1.00); however, those with papillary histology showed a higher propensity for FDG uptake than iodine uptake (WBS: n = 7/18, 39%; FDG: n = 16/18, 89%; *p* = 0.03) (Figure 4B,D). No significant differences were observed in bone uptake in follicular (WBS: n = 13/13, 100%; FDG: n = 10/13, 77%; *p* = 0.25) or papillary (WBS: n = 1/3, 33%; FDG: n = 3/3, 100%; *p* = 0.48) tumors (Figure 4C,D). Overall, uptake by histologic classification largely mirrored the uptake patterns of molecular classification.

### 4.3. Agreement between Imaging Modalities

We determined the agreement between imaging modalities on an individual patient level (see Section 2).

**Figure 5:**
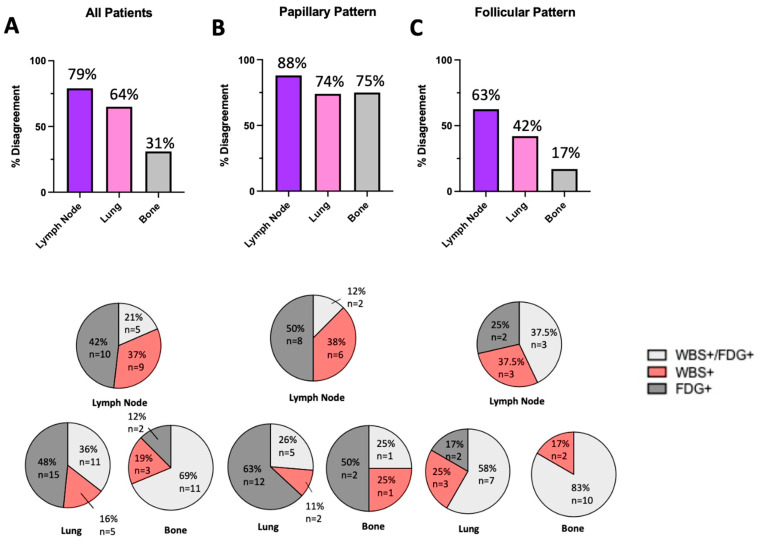
Agreement between imaging modalities by papillary or follicular pattern. Agreement is measured by percent discordance between WBS and FDG in patients who had positive uptake by at least one imaging modality. Discordance is inversely proportional to agreement. Below each bar chart, pie charts express the classification of each case into WBS+/FDG+, WBS+ only, and FDG+ only at each metastatic site. Discordance is the calculated sum of WBS+ only and FDG+ only. (**A**) In the entire cohort, percent discordant rates for lymph nodes (79%), lung (64%), and bone (31%). (**B**) In patients with papillary patterned thyroid cancer, lymph node (88%), lung (74%), and bone (75%). (**C**) In patients with follicular patterned thyroid cancer, lymph node (63%), lung (42%), and bone (17%).

In this analysis, discordant findings are consistent with the previously described flip-flop phenomenon, while concordant positive findings are not. In considering the entire cohort, lymphatic tissues displayed the highest degree of discordance (79%), followed by lung (64%), and bone (31%) (Figure 5A). In the papillary pattern sub-cohort, discordance at lymph, lung, and bone metastatic sites increased to 88%, 74%, and 75%, respectively (Figure 5B). The opposite pattern was identified in the follicular pattern sub-cohort with decreased discordance compared with the entire patient population: lymph (63%), lung (42%), and bone (17%) (Figure 5C).

In considering agreement between modalities stratified by *BRAF* V600E mutation status, *BRAF* V600E+ tumors resemble the papillary pattern sub-cohort with high lung (79%) and bone (75%) discordance (Supplemental Figure S2B). *BRAF* V600E−tumors resemble the follicular pattern sub-cohort with lower discordance in in lymph node (80%), lung (33%), and bone (0%) (Supplemental Figure S2C). Similarly, higher discordance is seen among tumors with papillary histology (lymph node 87%, lung 72%, bone 67%) across all sites compared with follicular histology tumors (lymph node 67%, lung 54%, bone 23%) (Supplemental Figure S3B,C). Notably, discordance at lymph node was the highest amongst all three sites irrespective of integrated pattern, *BRAF* V600E status, and histology.

## 5. Discussion

In this retrospective cohort study, we interrogate the effect of molecular subtype, histologic classification, and metastatic site on the flip-flop phenomenon in patients with metastatic DTCs. Our analysis reveals that the flip-flop phenomenon is not universally applicable across DTC molecular subtypes, histology, or metastatic sites.

Consistently with previous studies of BVL subtypes exhibiting greater dedifferentiation, RAI refractoriness, and tumor aggressiveness than RL subtypes [24,25,26,27,28], we find that tumors with papillary pattern, *BRAF* V600E mutation, and papillary histology have a higher propensity for FDG uptake compared with follicular pattern, *BRAF* V600E−, and follicular histology tumors, particularly in lung metastases. Previous studies support the link between molecular status, histology, and radiotracer avidity. For example, Ha et al. identified that the majority of RAI-negative and recurrent DTC were *BRAF* V600E+ and featured metastatic FDG avidity [38]. Soe et al. found that PTC with papillary architecture on histology were enriched in a cohort of non-iodine avid disease, further supporting the idea that BVL tumors are much more likely to be FDG avid [25]. Given that the majority of the papillary histology cohort consisted of *BRAF* V600E+ tumors (17/24, 71%) compared with that of the follicular cohort (3/22, 14%), it is not surprising that tumors with papillary histology largely behave like the BVL population and those with follicular histology are more congruent with the RL population by radiotracer imaging [28]. Although not reaching the level of statistical significance and limited by the cohort size in our study, *TERT*+ tumors behaved similarly as papillary and *BRAF* V600E+ tumors. Several studies have shown that *BRAF* and *TERT* promoter mutations promote the loss of RAI uptake, function cooperatively, and may adopt a more dedifferentiated phenotype than RL tumors [4,24,25,26,27]. Our results are therefore consistent with previous studies tying molecular status and histologic classification to dedifferentiation [24,26,27] and allow us to form two broader groups with internally consistent uptake patterns: (1) *BRAF* V600E+/*TERT*+/papillary histology and (2) *BRAF* V600E−/*TERT*−/follicular histology.

Our study adds to the existing literature by analyzing radiotracer uptake across common DTC metastatic sites. By combining information from histology or molecular subtype with metastatic site, our results may facilitate the selection of an appropriate radiotracer for the surveillance of tumor recurrence or progression. A summary of potential imaging recommendations is presented in Figure 5. For all molecular and histologic subtypes, the low agreement between both modalities at lymph node metastases renders both WBS and FDG-PET important for surveillance since neither modality may be sufficiently sensitive to reliably detect lymph node metastasis. In lung metastasis, papillary pattern, *BRAF V600E*+*,* and papillary histology tumors featured low agreement with strong FDG avidity such that FDG-PET may be considered the primary imaging modality. In contrast, for follicular pattern, *BRAF* V600E−, and follicular histology tumors, higher agreement and roughly equivalent RAI and FDG uptake renders either imaging modality appropriate. A previous study involving 83 patients with DTC lung metastases found that 30% (25/83) of patients had lung FDG uptake, while 55% (46/83) had lung radioiodine avidity, although this study did not differentiate follicular histology and PTC-FV from classical PTC, nor provide molecular correlates [39]. A population of tumors enriched in PTC-FV, follicular, or RL tumors may bias the cohort towards more differentiated phenotypes and thus higher rates of RAI avidity. In our study, FDG and RAI uptake was equivalent in bone for follicular pattern, *BRAF* V600E−, or follicular histology with high agreement for either imaging modality, an observation previously noted and attributed to the presence of mixed differentiated and dedifferentiated tumor tissues [40]. We observed lower agreement in bone metastasis for papillary pattern, BRAF V600E+, or papillary histology tumors which could represent a propensity for these tumors to be dedifferentiated, but our sample size is limited.

**Figure 6:**
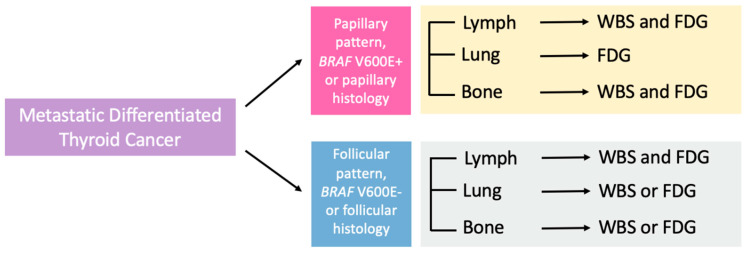
Schematic of recommended initial imaging modalities by molecular classification and histology.

Beyond guiding the imaging modality for surveillance, our study may help stratify metastatic DTC patients who could benefit from alternative therapies rather than empiric RAI ablation (Figure 6). We show that increased FDG avidity is associated with the *BRAF* V600E+/*TERT*+ molecular subtypes and papillary histology which, in turn, is indicative of dedifferentiated tumor tissue, increased aggressiveness, RAI insensitivity, and a worse prognosis [9,10,11,13,21,36]. For example, a patient with a BVL primary tumor and lung metastasis is much less likely to have iodine avid disease and tumor tissue capable of concentrating iodine. Such a patient may first benefit from iodine “resensitization” therapy with kinase inhibitors [31,32,33,34,35,41] prior to RAI ablation therapy. An interval follow-up with FDG-PET would be recommended given that FDG avidity is an independently poor prognostic factor in metastatic DTCs. In contrast, a patient with bone metastasis, regardless of subtype, may benefit from a combination of RAI and alternative therapies given that our radiotracer imaging findings are consistent with a mixture of differentiated states in bone metastasis.

The limitations of our study largely derive from cohort size, particularly with patients who have been explicitly tested for *TERT* promoter, the asynchronous imaging timeline (albeit limited to less than a 1-year inclusion criteria), and the potential for metastases to drift from the molecular signatures of the primary tumor.

Our study highlights the heterogeneous nature of the flip-flop phenomenon and identifies molecular subtype, histologic classification, and metastatic site as key variables for discrepancies in radiotracer uptake in metastatic DTCs. This knowledge of uptake patterns may be harnessed to guide the choice of appropriate modality for tumor surveillance, medical treatment, and the assessment of treatment response.

## Figures and Tables

**Table 1:** Patient characteristics, tumor histology, and molecular type.

Characteristics	Value
Number of patients (N)	46
Female	24 (52%)
Male	22 (48%)
Age at imaging [median (interquartile range)]	63 (55–73%)
Prior RAI (% of cohort)	12 (26%)
**Primary Histology (% of cohort)**	
Papillary (Tall, Classical, Sclerosing, Unknown Subtypes)	24 (52%)
Follicular (Follicular, Papillary-Follicular, Mixed Subtypes)	22 (48%)
**Histology by Subtype (% of cohort)**	
Papillary-Follicular (PTC-FV)	9 (20%)
Papillary-Tall	4 (9%)
Papillary Classical	13 (28%)
Papillary-Mixed	3 (7%)
Papillary-Sclerosing	1 (2%)
Papillary-Unknown	6 (13%)
Follicular (FTC)	10 (22%)
**Integrated Classification (% of cohort)**	
Papillary Pattern	27 (59%)
Follicular Pattern	19 (41%)
**Tumor Molecular Characteristics**	
BRAF V600E+	20 (44%)
BRAF V600E−	9 (20%)
BRAF V600E Untested	17 (37%)
	
RAS Mutation+	5 (11%)
RAS Mutation–	9 (20%)
RAS Untested	32 (70%)
	
TERT Mutation+	10 (22%)
TERT Mutation–	4 (9%)
TERT Untested	32 (70%)
**Metastatic Uptake (on either WBS or FDG)**	
Lymph node (% of cohort)	24 (52%)
Lung (% of cohort)	31 (67%)
Bone (% of cohort)	16 (35%)

RAI: radioactive iodine ablative therapy; PTC-FV: papillary thyroid cancer follicular variant; FTC: follicular thyroid cancer; WBS: radioactive iodine whole body scan; FDG: ^18^Fluorodeoxyglucose-PET; TERT: telomerase reverse transcriptase promoter.

## Data Availability

Data are available upon request and correspondence with Robert R. Flavell, MD, PhD at robert.flavell@ucsf.edu.

## Supplementary Materials

The following supporting information can be downloaded at: https://www.mdpi.com/article/10.3390/jcm13133963/s1, Figure S1: WBS and FDG uptake stratified by *TERT* promoter mutation status. Figure S2: Agreement between imaging modalities by *BRAF* status. Figure S3: Agreement between imaging modalities by histology. Table S1: Per-patient histology, classification, tumor genetics, and consensus uptake.

## Abbreviations

BVL	*BRAF* V600E-Like
DTC	Differentiated thyroid cancer
FDG	^18^Fluorodeoxyglucose
FP	Follicular pattern
MAPK	Mitogen-activated protein kinases
PET	Positron emission technology
PP	Papillary pattern
RAI	Radioactive iodine
RL	*RAS*-like
TERT	Telomerase reverse transcriptase
WBS	Whole body scan
