# Supplementary material for: Comparative Uptake Patterns of Radioactive Iodine and [18F]-Fluorodeoxyglucose (FDG) in Metastatic Differentiated Thyroid Cancers
Source: J Clin Med. 2024 Jul 6;13(13):3963. doi: 10.3390/jcm13133963 (PMC11242608; doi:10.3390/jcm13133963)

Supplementary Table S1: Per patient histology, classification, tumor genetics, and consensus uptake. Of note, 4 patients, Patient ID 22, 24, 25, and 26, featured metastases at sites other than lung, lymph, and bone.

Patient ID	Histology (DTC subtype)	Classification (Pattern)	BRAF V600E	RAS	TERT	Panel Testing	Additional Gene Mutations (if known)	Lung RAI	Lung FDG	Lymph RAI	Lymph FDG	Bone RAI	Bone FDG
1	P (Follicular)	PP	Y	NA	NA	NP		0	1	0	1	1	0
2	P (Tall)	PP	Y	NA	NA	NP		0	1	0	1	NI	NI
3	P (Classical)	PP	Y	NA	NA	NP		0	1	0	1	NI	NI
4	P (Classical)	PP	Y	NA	NA	NP		0	1	NI	NI	NI	NI
5	P (Classical)	PP	Y	NA	NA	NP		0	1	1	1	NI	NI
6	P (Classical)	PP	Y	N	Y	Y	None	1	1	0	1	NI	NI
7	P (Classical)	PP	NA	NA	NA	NP		NI	NI	1	0	NI	NI
8	P (Classical)	PP	NA	NA	NA	NP		NI	NI	0	1	NI	NI
9	P (Mixed)	FP	NA	NA	NA	NP		0	1	NI	NI	NI	NI
10	P (NA)	PP	NA	NA	NA	NP		1	0	1	0	NI	NI
11	P (Mixed)	FP	NA	NA	NA	NP		1	0	1	0	NI	NI
12	P (NA)	PP	N	N	N	Y	CCDC6-RET fusion	1	1	1	0	NI	NI
13	P (Classical)	PP	Y	N	Y	NP		0	1	NI	NI	NI	NI
14	P (Classical)	PP	NA	NA	NA	NP		0	1	NI	NI	NI	NI
15	P (NA)	PP	NA	NA	NA	NP		0	1	NI	NI	NI	NI
16	P (Sclerosing)	PP	Y	NA	NA	NP		1	1	0	1	NI	NI
17	P (Tall)	PP	Y	NA	Y	NP		0	1	1	0	0	1
18	P (NA)	PP	Y	N	Y	Y	None	1	1	1	1	0	1
19	P (NA)	PP	Y	NA	NA	NP		0	1	0	1	1	1
20	P (Tall)	PP	Y	NA	NA	NP		NI	NI	1	0	NI	NI
21	P (Classical)	PP	Y	NA	NA	NP		1	0	NI	NI	NI	NI
22	P (Follicular)	PP	Y	NA	NA	NP		NI	NI	NI	NI	NI	NI
23	P (Classical)	PP	Y	NA	NA	NP		0	1	0	1	NI	NI
24	P (Classical)	PP	Y	NA	NA	NP		NI	NI	NI	NI	NI	NI
25	P (Classical)	PP	Y	NA	NA	NP		NI	NI	NI	NI	NI	NI
26	P (Mixed)	PP	Y	N	Y	Y	None	NI	NI	NI	NI	NI	NI
27	P (NA)	PP	Y	N	Y	NP		0	1	NI	NI	NI	NI
28	P (Tall)	PP	Y	NA	NA	NP		NI	NI	1	0	NI	NI
29	F	FP	NA	NA	NA	NP		NI	NI	NI	NI	1	1
30	F	FP	NA	NA	NA	NP		NI	NI	NI	NI	1	1
31	F	FP	NA	NA	NA	NP		0	1	NI	NI	1	0
32	F	FP	NA	NA	NA	NP		NI	NI	NI	NI	1	1
33	F	FP	NA	Y	NA	NP		NI	NI	NI	NI	1	1
34	F	FP	N	Y	Y	Y	BCORL1, MT-ND5 fusion, TP53 mutation	NI	NI	NI	NI	1	1
35	P (Follicular)	FP	NA	NA	NA	NP		1	1	1	1	NI	NI
36	P (Follicular)	FP	NA	NA	NA	NP		1	1	0	1	NI	NI
37	P (Follicular)	FP	N	NA	NA	NP		NI	NI	1	0	1	1
38	F	FP	N	N	Y	Y	BRAFV601K, NF2	0	1	0	1	NI	NI
39	F	FP	N	N	Y	Y	FGFR2-SHTN1 fusion, MT-ND5 fusion	1	1	1	1	1	1
40	F	FP	N	Y	Y	Y	None	1	1	1	0	1	1
41	P (Follicular)	FP	N	Y	N	Y	None	NI	NI	NI	NI	1	1
42	P (Follicular)	FP	N	Y	N	Y		1	0	NI	NI	NI	NI
43	P (Follicular)	FP	NA	NA	NA	NP		1	1	NI	NI	1	1
44	P (Follicular)	FP	NA	NA	NA	NP		1	0	NI	NI	1	0
45	F	FP	NA	NA	NA	NP		1	1	1	1	NI	NI
46	P (Classical)	PP	N	N	N	Y	MET, PAX8, FOSB	1	1	NI	NI	NI	NI

Supplemental Figure S1: WBS and FDG Uptake Stratified by *TERT* Promoter Mutation Status
WBS: radioactive iodine whole body scan, FDG: ^{18}Fluorodeoxyglucose-PET. **(A)** No significant differences were observed in lymphatic tissue metastatic radiotracer uptake in any cohort. **(B)** Significantly increased FDG over iodine uptake was observed in the entire cohort in lung tissue and, to a lesser extent, in the *TERT*+ sub-cohort (p = 0.08), but not in the *TERT*- sub-cohort. **(C)** No significant differences in radiotracer uptake were observed in bone in any cohort. **(D)** Table summary with proportions of patients which have metastases at any given site and proportions of patients that had FDG or WBS positivity. Asterisk (*) indicates p < .05, ns = not significant.

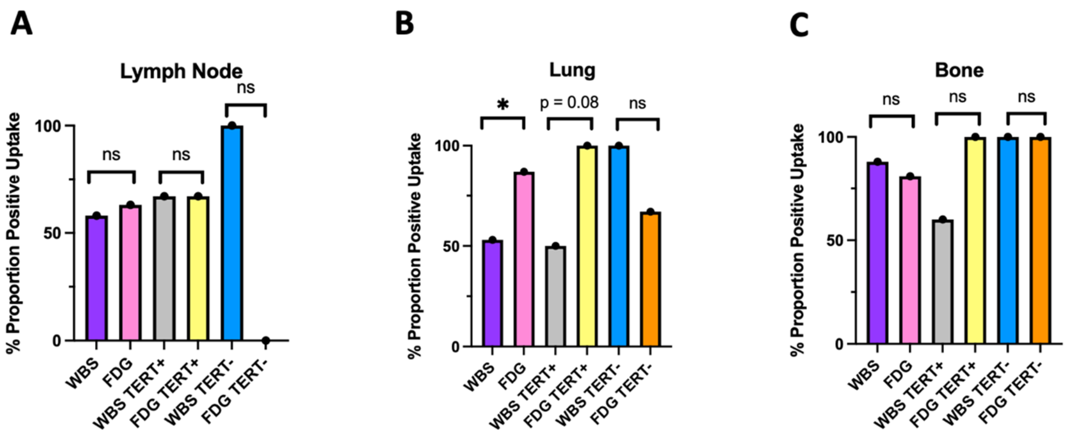

D

	**Lymph node** % positive cases/positive cases in cohort	**Lung** % positive cases/positive cases in cohort	**Bone** % positive cases/positive cases in cohort
WBS	14/23 (61%)	16/31 (52%)	14/16 (88%)
FDG	15/23 (65%)	26/31 (84%)	13/16 (81%)
WBS TERT+	4/6 (67%)	4/8 (50%)	3/5 (60%)
FDG TERT+	4/6 (67%)	8/8 (100%)	5/5 (100%)
WBS TERT-	1/1 (100%)	3/3 (100%)	1/1 (100%)
FDG TERT-	0/1 (0%)	2/3 (67%)	1/1 (100%)

Supplemental Figure S2: Agreement between imaging modalities by *BRAF* status.
WBS: radioactive iodine whole body scan, FDG: ^{18}Fluorodeoxyglucose-PET. Agreement is measured by percent discordance between WBS and FDG in patients who had positive uptake by at least one imaging modality. Discordance is inversely proportional to agreement. Below each bar chart, pie charts express the classification of each case into WBS + /FDG + , WBS+ only, and FDG+ only at each metastatic site. Discordance is the calculated sum of WBS+ only and FDG+ only. **(A)** In the cohort of patients with *BRAF* testing, percent discordant rates for lymph nodes (82%), lung (63%), and bone (33%). **(B)** In patients with *BRAF*+ thyroid cancer, lymph node (83%), lung (79%), and bone (75%). **(C)** In patients with *BRAF*- thyroid cancer, lymph node (80%), lung (33%), and bone (0%).

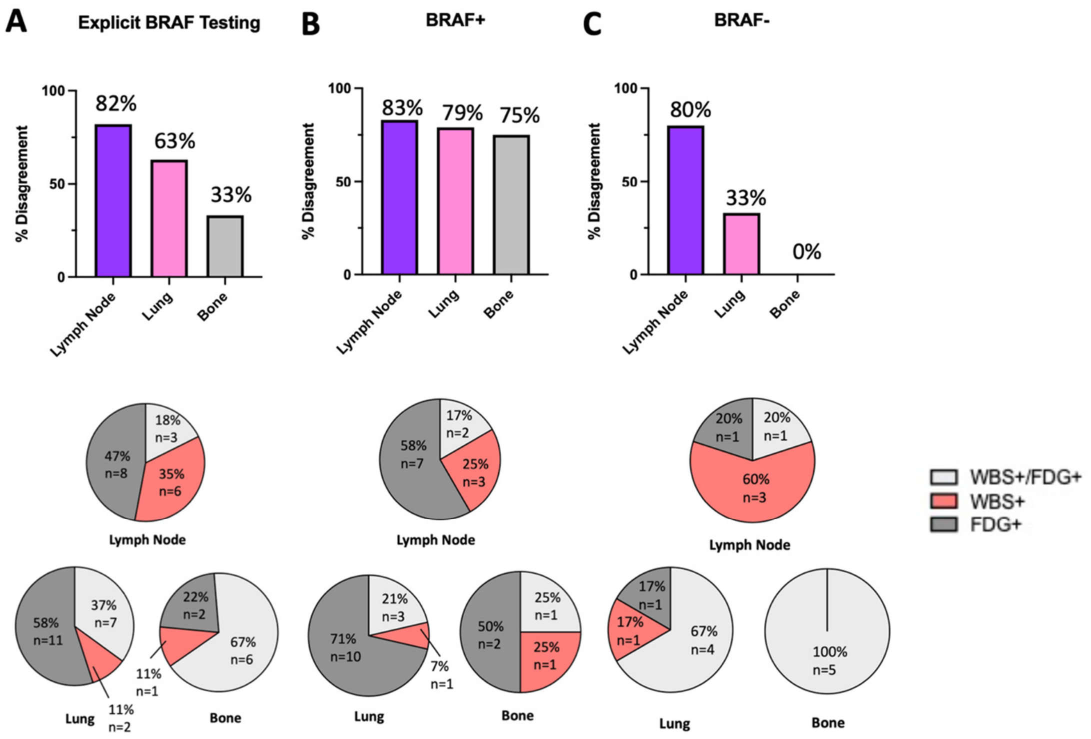

Supplemental Figure S3: Agreement between imaging modalities by histology.
WBS: radioactive iodine whole body scan, FDG: [18]Fluorodeoxyglucose-PET. Agreement is measured by percent discordance between WBS and FDG in patients who had positive uptake by at least one imaging modality. Discordance is inversely proportional to agreement. Below each bar chart, pie charts express the classification of each case into WBS + /FDG + , WBS+ only, and FDG+ only at each metastatic site. Discordance is the calculated sum of WBS+ only and FDG+ only. **(A)** In the entire cohort, percent discordant rates for lymph nodes (79%), lung (65%), and bone (31%). **(B)** In patients with papillary histology, lymph node (87%), lung (72%), and bone (67%). **(C)** In patients with follicular histology, lymph node (67%), lung (54%), and bone (23%).

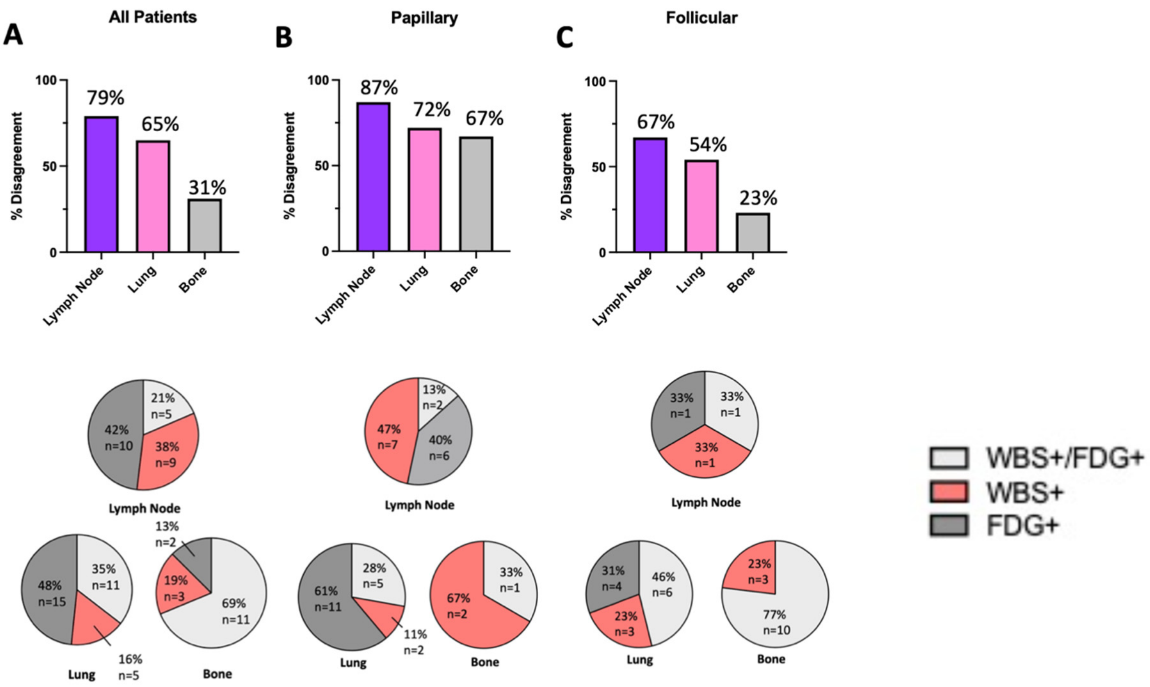

Supplement: Supplementary file 1 [file jcm-13-03963-s001.zip › jcm-3076554-supplementary.pdf]
